# Safety culture in the maternity unit of hospitals in Ilam province, Iran: a census survey using HSOPSC tool

**DOI:** 10.11604/pamj.2017.27.268.9776

**Published:** 2017-08-10

**Authors:** Nahid Akbari, Marzieh Malek, Parvin Ebrahimi, Hamid Haghani, Sanaz Aazami

**Affiliations:** 1 Faculty of Nursing and Midwifery, Iran University of Medical Sciences, Tehran, Iran; 2 Master student of Midwifery, Faculty of Nursing and Midwifery, Iran University of Medical Sciences, Tehran, Iran; 3 Department of Health Services Management, School of Health Management and Information Sciences, Health Management and Economics Research Center, Iran University of Medical Sciences, Tehran, Iran; 4 Department of Biostatistics, Faculty of Management and Information Technology, Iran University of Medical Sciences, Tehran, Iran; 5 Department of Nursing, Faculty of Nursing and Midwifery, Ilam University of Medical Science, Ilam, Iran

**Keywords:** Patient safety, patient safety culture, hospital survey on patient safety culture (HSOPSC), maternity units

## Abstract

**Introduction:**

Improving quality of maternal care as well as patients' safety are two important issues in health-care service. Therefore, this study aimed to assess the culture of patient safety at maternity units.

**Methods:**

This cross-sectional study was conducted among staffs working at maternity units in seven hospitals of Ilam city, Iran. The staffs included in this study were gynecologists and midwifes working in different positions including matron, supervisors, head of departments and staffs. Data were collected using the Hospital Survey on Patient Safety Culture (HSOPSC).

**Results:**

This study indicated that 59.1% of participants reported fair level of overall perceptions of safety and 67.1% declared that no event was reported during the past 12 months. The most positively perceived dimension of safety culture was teamwork within departments in view of managers (79.41) and personnel (81.10). However, the least positively perceived dimensions of safety culture was staffing levels.

**Conclusion:**

The current study revealed areas of strength (teamwork within departments) and weakness (staffing, punitive responses to error) among managers and personnel. In addition, we found that staffs in Ilam's hospitals accept the patient safety culture in maternity units, but, still are far away from excellent culture of patient safety. Therefore, it is necessary to promote culture of patient's safety among professions working in the maternity units of Ilam's hospitals.

## Introduction

Decreasing maternal mortality rate is recognized as an essential priority in health-care systems [1, 2]. Improving quality of maternal care as well as patients' safety are two important issues in health-care systems [1]. Recently, higher number of Iranian women are using maternal care due to increased birth rate [3]. International reports on maternal mortality rate has shown constant decreasing pattern during the last 15 years [4]. Interestingly, programs aimed at decreasing maternal mortality rate has always been a priority in the Iranian health system even, earlier than introducing continuous quality improvement model [3]. Some of these programs are promoting mother and child-friendly hospitals that offer maternal care using professional midwifery staffs. Nevertheless, maternal mortality rate in Iran is still high and reported at 20.3 deaths per 1000 live births. This number is shown to be higher in deprived provinces such as Ilam which is located in the west of Iran and has a shared border with Iraq [5]. There are some challenges in hospitals of deprived provinces including on-time emergency care, staff management, effective communication, teamwork and patients' safety in maternity units. Increasing quality of maternal care and enhancing level of maternal safety would lead to decreased maternal mortality rate [6–8]. Patients' safety refers to prevention of harms and errors imposed during providing care [9]. Promoting patients' safety and risk management are of great importance in maternity care units due to the fact that every procedure in this field will affect two individuals (both mother and baby) [10, 11]. Promoting effective patients' safety particularly in maternity care units might be influenced by organizational culture, teamwork, open communication, feedbacks, non-punitive responses to medical errors and common perception of staffs on patients' safety [12]. In order to reduce obstetrical errors, it is important to assess weakness and strengths of safety culture at maternity- units [13]. Therefore, this study aimed to assess safety culture in maternity units of the governmental hospital in Ilam, Iran.

## Methods

This cross-sectional study was conducted among staffs working at maternity units (including labor and delivery room, post-partum and operation room) in seven hospitals of Ilam University of Medical Sciences during year 2016. The staffs included in this study were gynecologists and midwifes working in several positions including managers, supervisors, head of departments and staffs. The total number of 350 staffs was invited to take part in the current study and 299 agreed to participate in the survey. Participants were informed about the aim, benefits and importance of the survey as well as their voluntary participation rights. Written informed consent was obtained prior to actual data collection. Data were collected using series of self-administrated questionnaires including socio-demographic characteristics and the Hospital Survey on Patient Safety Culture (HSOPSC). The socio-demographic and individual characteristics include age, gender, education, working hours per week, ward, organizational position, organizational tenure, work experiences in the current ward and type of employment. The Hospital Survey on Patient Safety Culture (HSOPSC) released by the Agency for Healthcare Research and Quality (AHRQ) to help hospitals assess the safety culture within their organization. The HSOPSC contains 42 items with 5 point Likert responses scale ranging from strongly disagree (1) to strongly agree (5). HSOPSC questionnaire contains both positively and negatively worded items. The average percent-positive score for each dimension was calculated and negative items were reverse scored prior to computing percent positive response. This questionnaire is designed to measure 12 dimensions of patient safety culture and these dimensions are: Supervisor/manager expectations and actions promoting safety (4 items), Organizational learning-continuous improvement (3 items), Teamwork within units (4 items), Communication openness (3 items), Feedback and communication about error (3 items), Non-punitive response to error (3 items), Staffing (4 items), Hospital management support for patient safety (3 items), Teamwork across hospital units (4 items), Hospital handoffs and transitions (4 items), Overall perceptions of safety (4 items), Frequency of event reporting (3 items) and Two outcome variables. The two outcome variables compose of “Overall perceptions of safety” and “Frequency of event reporting”. The HSOPSC questionnaire was validated in Persian language and shown a good validity and reliability to be used in Iran's hospitals [14]. However, the present study examined the internal consistency of Persian version of HSOPSC questionnaire and reported Cronbach alpha of 0.80. In addition, this study found that test re-tests reliability after 2 weeks was 0.78 which is considered as an acceptable reliability. Data analysis was conducted by SPSS version 20.

## Results

Mean age of participants in this study was 33.52 (SD= 6.37) years old (Table 1) and 84.1% were female with average 8.38 years organizational tenure and 5.63 years work experiences in the current position. Majority of participants (76.6 %) were midwives, 14.3% were working as managers, 31.9% in labor room, 24.6% in post-partum and 29.2% in operation room. Regarding educational status, 90.8% had bachelor, 4.8% had master and 4.4% were gynecologist. Less than half of the participants (48.6%) were employed on permanent contract. The average percent-positive responses across the 12 dimensions of safety culture among managers and personnel are described in Table 2. The most positively perceived dimension among managers was teamwork within departments (79.41), followed by organizational learning/ continuous improvement (76.47) and overall perception of safety (71.4). Similarly, the most positively perceived dimension among personnel was teamwork within departments (81.10). However, personnel perceived the teamwork within departments more positively compared to managers. The least positively perceived dimension was staffing among both managers (33.82) and personnel (26). Analysis of two outcome variables (Table 3) indicated that 59.1% of participants reported fair level of overall perceptions of safety and 67.1% declared that no event was reported during the past 12 months.

**Table 1:** Socio-demographic and work characteristics of respondents

	N	%
**Gender**		
Male	48	15.9
Female	253	84.1
**Department**		
Manager	43	14.3
Labor	96	31.9
Post-partum	74	24.6
Operation room	88	29.2
**Organizational position**		
Matron/Supervisor	51	17.1
Gynecologist	12	4
Nurse/Midwife	235	78.9
**Education**		
Bachelor	266	90.8
Master	14	4.8
Phd	13	4.4
**Age**	Mean	SD
	33.52	6.37
**Organizational tenure**	8.38	6.56
**Work experience in the current department**	5.63	5.58
**Working Hours per week**	49.53	14.78

**Table 2:** Average percent-positive responses across the 12 dimensions of safety culture among managers

	Managers		personnel	
Dimensions of safety culture	**Mean %**	**SD%**	**Mean%**	**SD%**
Overall Perception of Safety	74.1	26.43	73.70	23.19
Frequency of events reporting	45.9	43.11	56.93	42.65
Supervisor/manager expectations & actions promoting patient safety	72.54	28.39	45.40	12.30
Organizational learning/ continuous improvement	76.47	29.28	73.06	29.93
Teamwork within departments	79.41	29.87	81.10	24.23
Communication openness	62.09	36.52	58.80	31.97
Feedback and communication about error	64.05	32.55	66.53	34.51
Non-punitive response to error	35.29	32.93	35.60	29.86
Staffing	33.82	29.5	26.00	23.69
Hospital management support for patient safety	69.28	36.41	47.46	36.89
Teamwork across hospital departments	65.68	43.28	53.30	39.46
Hospital handoffs & transitions	57.84	39.84	65.20	34.18

**Table 3:** Average percent-positive scores for two outcome variables

Two outcome	N	%
**Patient safety Grade**		
Excellent	26	8.8
Very Good	64	21.6
Acceptable	175	59.1
Poor	31	10.5
**Number of events reported**		
No event	202	67.1
one to two events	44	14.6
three to five events	22	7.3
six to ten events	8	2.7
More than 21 events	1	0.3
No response	24	24

## Discussion

This study assessed culture of patient's safety in hospitals of Ilam University of Medical Sciences. Creating culture of patient safety requires assessment of safety culture using appropriate tool. This study measured culture of patient safety using HSOPSC questionnaire which is one of the best tools in this area [15]. The current study revealed areas of strength and weakness among managers and personnel. The most positively perceived dimension of safety culture was attributed to “teamwork within department” among both managers and personnel. It has been shown that “teamwork within department” is perceived as the most strength dimension of safety culture among several countries including Saudi Arabia [10], United Stated America and Turkey [16]. Teamwork approach allows staffs to work together, share the responsibilities and reduce medical errors. In addition, lack of inter-sectoral collaboration is a barrier in providing culture of patient safety [17]. However, our findings showed that personnel perceived the teamwork more positively compared to managers. This finding is consistent with previous evidence showing that different professions [18] and even individuals from a same professions category [19] may perceive the safety culture differently. Another finding from this study indicated that the least positively perceived dimension was “staffing” and “non-punitive response error”, respectively. However, positive perception toward “staffing” was higher among managers compared to personnel and “non-punitive response error” was almost equal between two groups. Similarly, previous finding from Saudi Arabia [10] indicated that two dimensions of safety culture namely: “staffing” and “non-punitive response error” were areas requiring improvement. These findings indicate that there are inadequate staffs to handle the work, staffs work in long hours and try to work in crisis mood to do too much work quickly. Organizations with insufficient number of employee face several consequences such work-overload, burnout, sleepless which in turn lead to lower quality of patient care [20, 21] .Work overload has been shown as a reason for reported drug errors [22].On the other hand, punitive response to errors is reported as a challenge for our participants. This finding is critical given the emerging importance of non-punitive response to medical errors during the last decades. Prevention, early diagnosis and effective management of medical errors are recognized as the priority in health-care systems [23]. Majority of our participants reported no events during the last 12 months which is in contrast to the high frequency of medical errors in developing countries [24]. A possible explanation for underestimated report of medical error could be the punitive response to error in health-care organizations [25].

## Conclusion

The results of this study showed acceptable levels of overall perception of safety culture among both managers and personnel with higher perception among managers. This finding implies that health-care workers in Ilam's hospitals accept the patient safety culture in maternity units, but still are far away from excellent culture of patient safety. Maternal wards are among very important units in each hospital, which require special attentions. Therefore, it is necessary to promote culture of patient's safety among professions working in the maternity units of Ilam's hospital. There are several limitations in this study, which need to be addressed. One of the limitations in this study was lack of evidences in maternity units to compare the results. Therefore, future studies are recommended to assess the culture of patient safety in maternal care units using HSOPSC questionnaire. Another limitation in this study is use of self-reported data.

### What is known about this topic

- International reports on maternal mortality rate has shown constant decreasing pattern during the last 15 years;
- Programs aimed at decreasing maternal mortality rate has always been a priority in the Iranian health system and some of these programs are promoting mother and child-friendly hospitals that offer maternal care using professional midwifery staffs;
- Promoting patients' safety and risk management are of great importance in maternity care units due to the fact that every procedure in this field will affect two individuals (both mother and baby).

### What this study adds

- This study assessed culture of patient's safety in hospitals of Ilam University of Medical Sciences for the very first time and revealed areas of strength and weakness among managers and personnel;
- The most positively perceived dimension of safety culture was attributed to “teamwork within department” among both managers and personnel. While, the least positively perceived dimension was “staffing” and “non-punitive response error”, respectively;
- Positive perception toward “staffing” was higher among managers compared to personnel and “non-punitive response error” was almost equal between two groups.

## Competing interests

The authors declare no competing interests.
